# CX3CR1-microglia mediates neuroinflammation and blood pressure regulation in the nucleus tractus solitarii of fructose-induced hypertensive rats

**DOI:** 10.1186/s12974-020-01857-7

**Published:** 2020-06-12

**Authors:** Chiu-Yi Ho, Yu-Te Lin, Hsin-Hung Chen, Wen-Yu Ho, Gwo-Ching Sun, Michael Hsiao, Pei-Jung Lu, Pei-Wen Cheng, Ching-Jiunn Tseng

**Affiliations:** 1 Department of Medical Education and Research, Kaohsiung Veterans General Hospital, Kaohsiung, 81300 Taiwan; 2 Institute of Biomedical Sciences, National Sun Yat-Sen University, Kaohsiung, 80424 Taiwan; 3 Section of Neurology, Kaohsiung Veterans General Hospital, Kaohsiung, 81300 Taiwan; 4 Center for Geriatrics and Gerontology, Kaohsiung Veterans General Hospital, Kaohsiung, 81300 Taiwan; 5 Shu-Zen Junior College of Medicine and Management, Kaohsiung, 82144 Taiwan; 6 Division of General Internal Medicine, Department of Internal Medicine, Kaohsiung Medical University Hospital, Kaohsiung Medical University, Kaohsiung, 80708 Taiwan; 7 Division of Internal Medicine, School of Medicine, College of Medicine, Kaohsiung Medical University, Kaohsiung, 80708 Taiwan; 8 Department of Anesthesiology, Kaohsiung Medical University Hospital, Kaohsiung, 80708 Taiwan; 9 Department of Anesthesiology, Faculty of Medicine, College of Medicine, Kaohsiung Medical University, Kaohsiung, 80708 Taiwan; 10 Genomics Research Center, Academia Sinica, Taipei, 11529 Taiwan; 11 Institute of Clinical Medicine, National Cheng-Kung University, Tainan, 70101 Taiwan; 12 Department of Medical Research, China Medical University Hospital, China Medical University, Taichung, 40402 Taiwan

**Keywords:** Nucleus tractus solitarii, Fractalkine, CX3CR1, Hypertension, Inflammation

## Abstract

**Background:**

Inflammation is a common pathophysiological trait found in both hypertension and cardiac vascular disease. Recent evidence indicates that fractalkine (FKN) and its receptor CX3CR1 have been linked to inflammatory response in the brain of hypertensive animal models. Here, we investigated the role of CX3CR1-microglia in nitric oxide (NO) generation during chronic inflammation and systemic blood pressure recovery in the nucleus tractus solitarii (NTS).

**Methods:**

The hypertensive rat model was used to study the role of CX3CR1-microglia in NTS inflammation following hypertension induction by oral administration of 10% fructose water. The systolic blood pressure was measured by tail-cuff method of non-invasive blood pressure. The CX3CR1 inhibitor AZD8797 was administered intracerebroventricularly (ICV) in the fructose-induced hypertensive rat. Using immunoblotting, we studied the nitric oxide synthase signaling pathway, NO concentration, and the levels of FKN and CX3CR1, and pro-inflammatory cytokines were analyzed by immunohistochemistry staining.

**Results:**

The level of pro-inflammatory cytokines IL-1β, IL-6, TNF-α, FKN, and CX3CR1 were elevated two weeks after fructose feeding. AZD8797 inhibited CX3CR1-microglia, which improved the regulation of systemic blood pressure and NO generation in the NTS. We also found that IL-1β, IL-6, and TNF-α levels were recovered by AZD8797 addition.

**Conclusion:**

We conclude that CX3CR1-microglia represses the nNOS signaling pathway and promotes chronic inflammation in fructose-induced hypertension. Collectively, our results reveal the role of chemokines such as IL-1β, IL-6, and TNF-α in NTS neuroinflammation with the involvement of FKN and CX3CR1.

## Introduction

Cardiovascular disease is a complicated condition that affects metabolism and blood pressure [1, 2]. Epidemiological study showed that unhealthy dietary habit contributes to increased blood pressure, hyperglycemia, hyperlipidemia, and neuroinflammation [3]. Despite recent studies showing that excessive fructose consumption leads to pro-inflammatory response and microglia activation, the role of pro-inflammatory factor in the nucleus tractus solitarii (NTS) is not well-understood [4, 5]. In addition, we previously showed that the brainstem NTS, part of the brain which integrates signals from the peripheral carotid sinus and aortic arch to regulate systemic blood pressure, decreases production of nitric oxide (NO) during fructose-induced hypertension [6]. However, the underlying mechanism involving NO during chronic inflammation in the NTS is not yet clarified. Therefore, it is essential to investigate the role of FKN and its receptor CX3CR1 after hypertension induced by fructose.

C-X3-C motif chemokine receptor 1 (CX3CR1) is a chemokine receptor that binds to its ligand C-X3-C motif chemokine ligand 1 (CX3CL1), also known as fractalkine (FKN), and is found in the leukocytes, brain, spinal cord, and retina [7, 8]. Both FKN and CX3CR1 have been detected in vivo in the brainstem NTS. CX3CR1 is characterized as microglia biomarker. According to Bhaskar et al., CX3CR1 is the potential target for studying metabolic syndrome [9, 10]. The FKN–CX3CR1 interaction is associated with crosstalk between neurons and microglia. Previous study has suggested that CX3CR1 may be involved in neurodegenerative diseases such as multiple sclerosis [11], Alzheimer’s disease [5], spinal cord injury, and traumatic brain injury [12]. Ruchaya et al. reported that FKN microinjection produces cardiovascular response in the NTS of normal rats [13]. However, the function of CX3CR1 during neurogenic hypertension progression in the NTS is ambiguous. On the other hand, NO is a gas molecule that is involved in various functions in the dorsal brainstem NTS such as vasodilation, baroreflex, sympathetic nerve activity, and most importantly blood pressure maintenance [14]. It is essential that the autonomic response to combine afferent signals be integrated from the peripheral nervous system for optimal adjustment of sympathetic activity. In the clinical aspect, overactivity of the sympathetic nervous system contribute to the development of hypertension [15]. This study aims to examine the NTS cardiovascular effect and whether increased pro-inflammatory cytokines such as CX3CR1 has impact on NO production.

There is increasing evidence that inflammation in the brain is associated with excessive fructose intake. Such inflammatory response poses a threat to the brain and microglia cells which reside in the brain that act upon the threat to clear debris and repair damaged neural tissue. However, chronic inflammation in the brain activates microglia cells to release pro-inflammatory cytokines that are harmful to brain tissue [16, 17]. Furthermore, fructose, in particular, induces hypertension in vivo which strongly correlates with chronic brain inflammation [18–20]. Xu et. al. reported that fructose increases blood pressure, and elevates transcription of pro-inflammatory cytokines, IL-1β, IL-6, and TNF-α in the brain after 8 weeks of fructose consumption [5].

Our previous results suggest that fructose reduces central NO production, increases systemic blood pressure and renal sympathetic nerve activity [14, 21, 22]. The signaling pathway, PI3K–Akt–ERK1/2–nNOS (neuronal nitric oxide synthase) pathway, is shown to regulate nNOS phosphorylation in the NTS [23]. Additionally, we found that fructose disrupted the Akt–ERK1/2–nNOS signaling pathway [6, 21]. Short-term or 2 weeks of fructose feeding increases TNF-α in the brain [4]. We previously reported that prolonged fructose feeding time period induces blood pressure [22]. These results support the idea that blood pressure increase triggered by dietary fructose is closely linked to pro-inflammatory cytokines in the brain. We therefore speculate that CX3CR1-microglia may affect NO production to modulate NTS inflammation, and selective CX3CR1 inhibitor AZD8797 may be able to treat the NTS pro-inflammatory response. We observed that pro-inflammatory cytokines and NO production were suppressed through abolishing the Akt–ERK1/2–nNOS signaling pathway. Finally, microglia activation in the NTS requires CX3CR1 to promote fructose-induced hypertension progression.

## Methods

### Animal care

All procedures were reviewed and approved by the Institute of Animal Care and Use Committee at the Kaohsiung Veterans General Hospital (VGHKS, Kaohsiung, Taiwan). Wistar-Kyoto rats (WKY) were purchased from National Laboratory Animal Center (NLAC, Taipei, Taiwan), housed in the VGHKS animal center with light-controlled cycle of 12-h light and 12-h darkness.

### Experimental design

WKY animals were divided into five groups. Each consisted of six to eight animals in total: (1) control rats received tap water (Ctrl); (2) hypertensive rats received 10% fructose water for 2 or 4 weeks (F2w, F4w); (3) vehicle-control rats were fed with tap water for 2 weeks, followed by osmotic pump implantation (14 days, 0.5 μL/h) filled with 30% 2-hydroxypropyl-β-cyclodextrin (HP-β-CD) (Santa Cruz, Dallas, TX, USA) and continued tap water feeding simultaneously in the subsequent 2 weeks (V-Ctrl); (4) vehicle-fructose rats received 10% fructose water for 2 weeks, followed by osmotic pump implantation (14 days, 0.5 μL/h) filled with 30% 2-hydroxypropyl-β-cyclodextrin (HP-β-CD) and continued 10% fructose water simultaneously in the subsequent 2 weeks (V-Fru); (5) finally, the fructose-AZD8797 rats received 10% fructose water for 2 weeks, followed by osmotic pump implantation (14 days, 0.5 μL/h) filled with AZD8797 (Axon Medchem, Groningen, Netherlands) solution and continued 10% fructose water simultaneously in the subsequent 2 weeks (Fru-AZD).

### Non-invasive blood pressure (NIBP) measurement

The systolic blood pressure (SBP) was measured by using the tail-cuff method (CODA, Kent Scientific, Torrington, CT, USA). Operating procedures were carried out according to the manufacturer’s manual. Briefly, the WKY rats were trained in a rodent holder and placed on the warm plate (35 °C) for three times before the experiment began. Measurements were taken 15 times, restricted to 40 min and below for the entire procedure, and systolic blood pressure data were collected at the end of the experiment. To prevent blood pressure fluctuation as a result of circadian rhythms, the data were collected between 09:00–12:00 am, coordinated universal time with +08:00 offset for Taiwan time zone.

### Cerebrospinal fluid (CSF) and serum sample collection and measurement

After a 16-hr fast, the rats were anesthetized with isoflurane (2% mixed with O_2_), and blood samples were collected using serum separation tubes. To collect serum, the tubes were centrifuged (3000 × *g*) at 4 °C for 10 min. The CSF was collected in a 1.5-mL Eppendorf tube from the fourth ventricle through an insulin needle. The tubes were centrifuged (300 × *g*) at 4 °C for 10 min to remove residual blood. Immediately, all samples were dispensed and frozen at − 80 °C for biochemical assays. All serum parameters were measured by VITROS® 350 Chemistry System (Ortho Clinical Diagnostics, New Brunswick, NJ, USA).

### Intracerebroventricular administration procedure

The three animal groups: V-Ctrl, V-Fru, and Fru-AZD underwent intracerebroventricular surgery. The osmotic pump (Alzet, Cupertino, CA, USA) which was filled with AZD8797solution, vehicle solution 30% 2-hydroxypropyl-β-cyclodextrin (HP-β-CD), was immersed in normal saline for 16 h at 4 °C prior to surgery. During anesthetization, Atropine 0.05 mg/kg, Zolile 40 mg/kg, and Xylazine 10 mg/kg were placed on the stereotaxic instrument; the head was fixed with the cranium exposed. The position of the injection was located 1.5 mm lateral and caudal 0.8 mm from the bregma. After surgery, the rat tail systolic blood pressure was measured every week.

### Immunoblot analysis

The brain was excised and the NTS regions (approximately 20 mg) were separated according to the rat brain in stereotaxic coordinates based on Paxinos and Watson, sixth edition in 2007 [24]. Briefly, the NTS was dissected by micropunch (1 mm inner diameter) from 1-mm-thick brainstem slice at the level of the obex under the microscope. To collect total protein, NTS tissue was homogenized using T-PER (Thermo Fisher, Waltham, MA USA) containing protease and phosphatase inhibitors cocktail at 4 °C. Protein was quantitatively analyzed with Coomassie R-250 (Thermo Fisher, Waltham, MA USA), subjected to 4–20% SDS gradient gel electrophoresis, transferred to PVDF membrane. The membranes were blocked with 5% BSA in TBS/Tween-20 buffer (10 mM Tris, 150 mM NaCl, 0.1% Tween 20, pH 7.4), incubated with anti-p-Akt^S473^ (Cell signaling, 9271), anti-Akt (Cell signaling, 9271), anti-p-eNOS^S1177^ (BD, 612393), anti-eNOS (BD, 610297), anti-p-nNOS^S1416^ (Abcam, ab5583), anti-nNOS (Millipore, 07-571), anti-p-ERK1/2^T202/Y204^ (Cell signaling, 9101), anti-ERK1/2 (Cell signaling, 9102), or β-actin (Millipore, MAB1501) antibody at 1:1000 dilution in phosphate-buffered saline (PBS) Tween-20 with 5% BSA at 4 °C overnight. Peroxidase-conjugated anti-mouse or anti-rabbit antibody (1:5000) was used as the secondary antibody. The proteins were visualized using enhanced chemiluminescence (ECL), Pico plus detection kit (Thermo Fisher, Waltham, MA, USA), and film. The films were captured by ChemiDoc™ MP Imaging System (Bio-Rad, Hercules, CA, USA) and analyzed with Image Lab™ Software (Bio-Rad, Hercules, CA, USA).

### Analysis of NO concentration in the NTS

To determine NO concentration in the groups V-Ctrl, V-Fru, and Fru-AZD, the NTS protein lysate was deproteinized using a Microcon YM-30 filter unit (Millipore, Darmstadt, GmbH) based on a previous method [6]. The total amount of NO_*x*_ in the samples were determined using modified chemiluminescence-based procedure and Sievers Nitric Oxide Analyzer purge system (NOA 280i,Sievers Instruments, Boulder, CO, USA) [25].The sample (10 μL) was injected into reflux column containing 0.1 mol/L VCl_3_ in 1 mol/L HCl at 90 °C to reduce nitrates and nitrites into NO. The NO_*x*_ was then combined with O_3_ produced by the analyzer to form NO_2_. The emission resulting from the excited NO_2_ was detected by a photomultiplier tube and digitally recorded (mV). The values were then interpolated to standard curve of concurrently determined NaNO_2_ concentrations. The measurements were recorded in triplicate for each sample. The NO_*x*_ levels measured were corrected for the NTS protein concentration of the rats.

### Enzyme-linked immunosorbent assay (ELISA)

The NTS of the brainstem were sectioned and homogenized with T-PER (Thermo Fisher, Waltham, MA, USA) containing protease and phosphatase inhibitors cocktail at 4 °C. The total protein was harvested by grinding and centrifugation. The total protein content was quantitatively analyzed by Coomassie R-250 (Thermo Fisher, Waltham, MA, USA). The concentration of IL-1β, IL-6, TNF-α, and fractalkine of serum, CSF, or NTS protein lysate were measured by ELISA kit, performed according to the manufacturer’s instruction (Cloud-Clone Corp, Katy, TX, USA). Expression values were detected by Anthos Zenyth 200rt Microplate Reader (Biochrom, Cambridge, UK). The final values were calculated and normalized to NTS protein mass.

### Immunofluorescent staining analysis

The Ctrl, F2w, and F4w animals were perfused with saline, followed by 4% paraformaldehyde solution. The brainstem was harvested and immersed in 30% sucrose solution until it was sunken to the bottom of the tube, and this procedure was repeated once. Brain stem sections (5 μm) were blocked with 5% bovine serum albumin and 0.3% Triton X-100 for 30 min at room temperature, incubated in primary antibody anti-Iba-1 (Wako, 019-19741) for 16 h at 4 °C. After PBS wash, the sections were incubated in Alexa Flour 488 Goat anti-rabbit IgG (Thermo Fisher Scientific, Waltham, MA, USA) for 1 h under room temperature. The tissues were mounted in VECTASHIELD mounting medium containing DAPI (Vector Labs, Burlingham, CA, USA). The sections were analyzed under LSM 800 laser scanning mode of confocal microscope (Carl Zeiss MicroImaging, Jena, GmbH). The images were acquired using 40 x magnification (objective: Plan-APO 40 x/1.30 Oil DIC (UV) VIS-IR ), image matrix of 1024 × 1024 pixel, pixel scale 0.156 × 0.156 μm, and a depth of 8 bit. Z-stacked images were collected with 0.33-μm slice distance for 15 slices in total.

### Immunohistochemistry staining analysis

The brain stem sections (5 μm) were blocked in 5% bovine serum albumin and 0.3% Triton X-100 for 30 min at room temperature, incubated in primary antibody anti-IL-1β (Proteintech, 16806-1-AP) and anti-IL-6 (Proteintech, 21865-1-AP) in primary antibody diluent (ScyTek laboratories, Logan, UT, USA) for 16 h at 4 °C. After PBS wash, the sections were incubated in Novolink Polymer solution (Leica Biosystems, Nussloch, GmbH) for 10 min under room temperature. The tissues were stained in DAB chromogen at room temperature, analyzed by Olympus BX51 microscope (Olympus Tokyo, Japan) and Image Browser (Carl Zeiss, MicroImaging, Jena, GmbH).

### Statistical analysis

All data were expressed as mean ± SEM at least three independent experiments. IBM SPSS Statistics 20 was used in this study. The blood pressure (BP) measurements (fructose-treated and no-treatment groups) were analyzed by one-way ANOVA for repeated measurements and Bonferroni’s post-hoc tests. One-way ANOVA with Scheffe’s post-hoc comparison was applied to immunoblotting and immunohistochemistry stain. *P* < 0.05 was considered statistically significant.

## Results

### Fructose consumption leads to higher blood pressure and central inflammation

Fructose feeding induces hypertension in rats, and data are analyzed at the second and fourth weeks. The systolic blood pressure, fasting blood glucose, high-density lipoprotein, and triglyceride content were increased in the fructose group compared to control. Pro-inflammatory cytokines IL-1β and IL-6 and TNF-α significantly increased in serum and NTS after fructose administration, but FKN showed no increase in the serum after 4 weeks of fructose feeding. Interestingly, FKN in the CSF and NTS increased after 2 weeks of fructose feeding (Table 1). These data demonstrated that fructose consumption leads to higher blood pressure and central inflammation.

**Table 1:** General characteristics of the fructose-induced WKY rats

Parameter/group	Control	Fructose 2 weeks	Fructose 4 weeks
Systolic blood pressure, mmHg	109.8 ± 1.8	125.1 ± 2.2***	144.5 ± 1.1***
Fasting serum glucose, mg/dL	85.3 ± 1.8	134.2 ± 4.9***	147.0 ± 4.0***
Fasting serum triglyceride, mg/dL	82.7 ± 1.2	155.3 ± 18.6***	177.8 ± 26.6***
Fasting serum dHDL, mg/dL	83.0 ± 1.6	75.5 ± 4.5*	71.5 ± 2.5*
Serum IL-1β, pg/mL	27.93 ± 5.13	49.97 ± 8.59*	74.90 ± 7.07*
Serum IL-6, pg/mL	3.44 ± 0.46	6.21 ± 0.24*	10.02 ± 1.69*
Serum TNF-α, pg/mL	25.82 ± 9.05	74.06 ± 5.62*	202.10 ± 17.82*
Serum fractalkine, ng/mL	0.38 ± 0.06	0.36 ± 0.04	0.43 ± 0.05
CSF fractalkine, ng/mL	1.20 ± 0.04	1.49 ± 0.07*	1.65 ± 0.03*
NTS fractalkine, ng/mg	4.06 ± 0.17	4.80 ± 0.36*	5.98 ± 0.38*
NTS IL-1β, pg/mg	6.53 ± 0.27	9.17 ± 0.32*	12.63 ± 0.83*
NTS IL-6, pg/mg	61.13 ± 6.30	115.48 ± 3.30*	150.87 ± 11.68*
NTSTNF-α, pg/mg	1.15 ± 0.24	4.67 ± 0.75*	12.17 ± 0.41*

Levels of systolic blood pressure; fasting serum glucose; fasting serum triglycerides; fasting serum dHDL; and quantitative ELISA of serum, CSF, or NTS areas for FKN, IL-1β, IL-6, and TNF-α are presented as mean ± SEM. One-way ANOVA with Bonferroni’s post-hoc was performed for statistical analysis.

*P < 0.05, ***P < 0.001 compared to control group (*n* = 6~8 per group)

### Microglia mediate blood pressure regulation in the NTS in fructose-induced hypertension

**Fig. 1:**
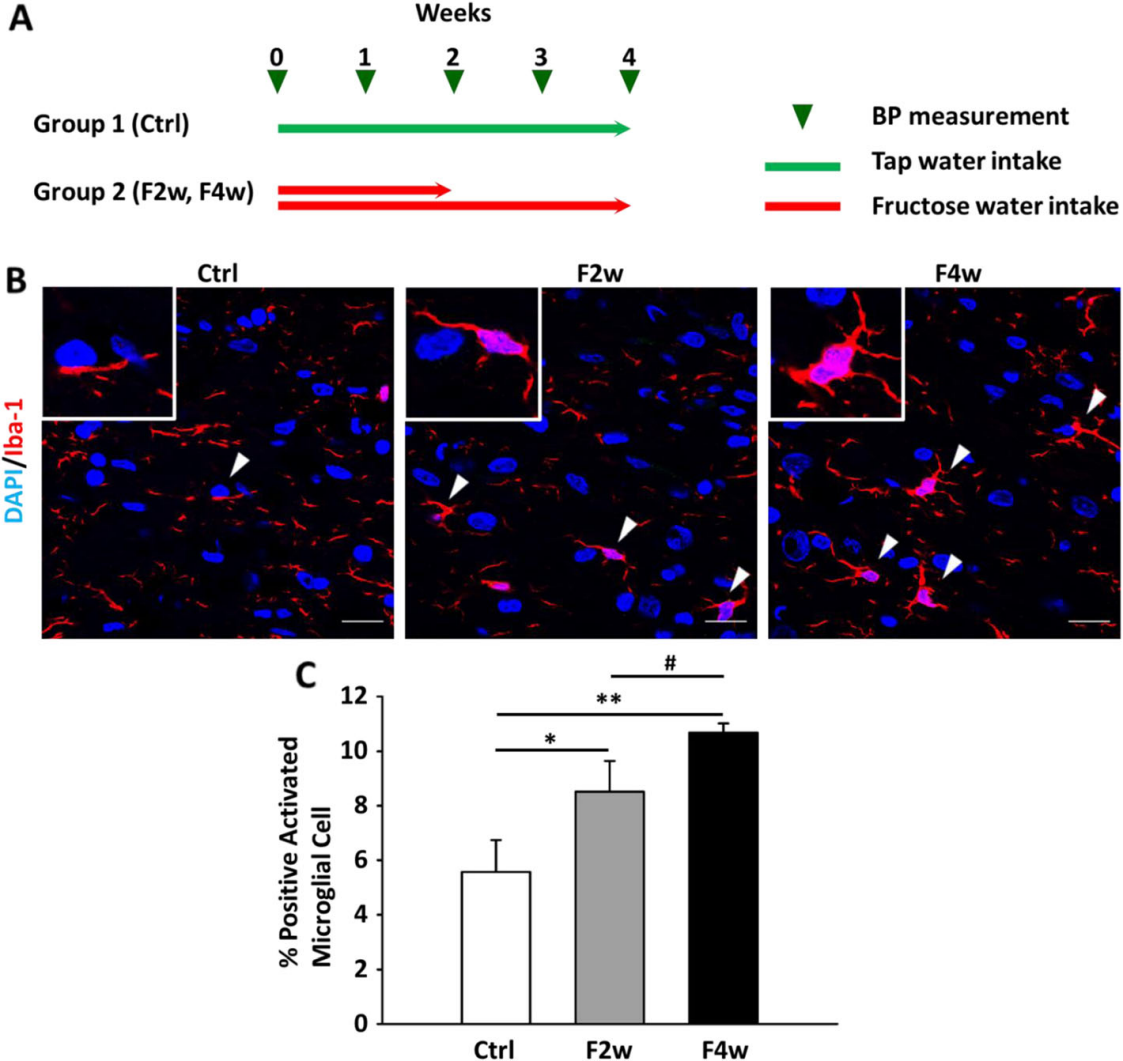
Quantitative immunofluorescent analysis of microglial marker in the NTS following feeding with 10% fructose. **a** Flowchart presented animal experimental design. **b** In situ qualitative analysis of the microglial marker Iba-1 by immunofluorescent staining. The arrowhead indicates activated microglial cells. The scale bar represents 20 μm. **c** Graphs depicting quantitative analysis of in situ positive cells in the NTS of WKY rats after fructose feeding. The percentage of the positive cells was determined by counting positive cells in each hemisphere of the NTS at 400 × magnification. The values are presented as mean ± SEM. One-way ANOVA with Bonferroni’s post-hoc was performed for statistical analysis.*P < 0.05, **P < 0.01 compared to the control group, #*P* < 0.05 compared to fructose feeding for 4 weeks (*n* = 6~8 per group)

Previously, CX3CR1 has been shown to be a putative microglial marker which was implicated to regulate blood pressure and heart rate [13]. In this study, we determined microglia activation through Iba-1 staining in NTS after fructose administration. The activated microglia showed stronger IBA-1 signal and shorten processes in the NTS after 2 or 4 weeks of fructose feeding (Fig. 1a). We quantified the NTS-activated microglia for fructose and control groups (Fig. 1b). Based on these observations, we speculate that microglia may have begun to intervene in the NTS, affecting blood pressure in the early stage of fructose-induced hypertension.

**Fig. 2:**
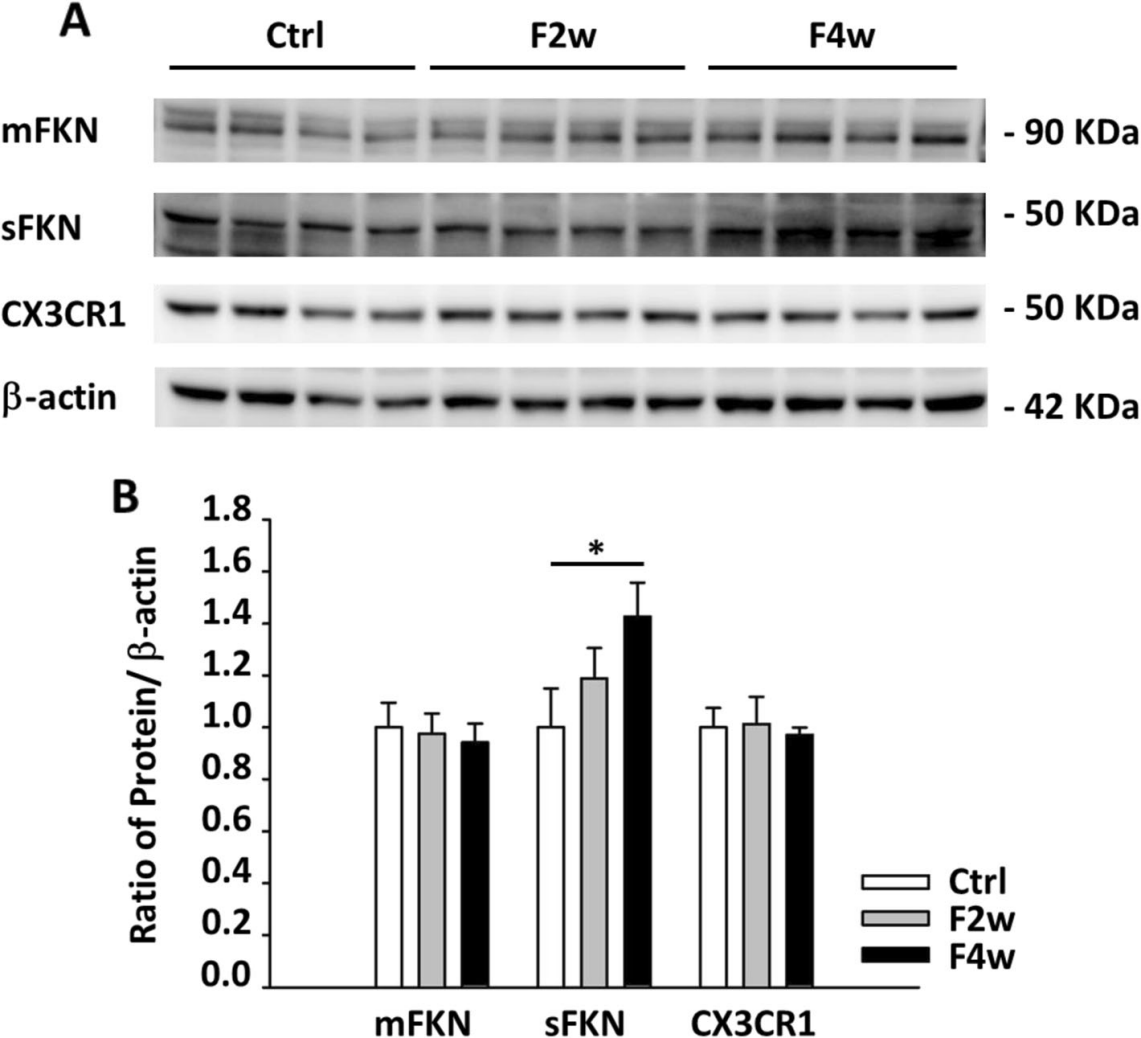
Semi-quantitative protein expression of membrane-anchored FKN, soluble FKN, and CX3CR1 in the NTS after fructose feeding for 2 or 4 weeks. **a** The immunoblotting of FKN in the fructose and control animals after 4 weeks of fructose feeding. **b** Graphs depicting semi-quantitative analysis of protein expression level in NTS of WKY rats after fructose feeding. The values are presented as mean ± SEM. One-way ANOVA with Bonferroni’s post-hoc was performed for statistical analysis. *P < 0.05 compared to the control group (*n* = 6~8 per group)

The fractalkine is a sole ligand that couples to CX3CR1, existing in either membrane-anchored or soluble form. The membrane-anchored FKN (mFKN), soluble FKN (sFKN), and CX3CR1 were analyzed by immunoblotting (Fig. 2a). After 4 weeks of fructose feeding, the sFKN was increased in the NTS but not mFKN and CX3CR1. Next, the level of sFKN in the NTS was significantly higher compared to control after 4 weeks of fructose feeding (Fig. 2b). As a result, we speculate that soluble FKN may have begun to stimulate microglia activation in the NTS after fructose consumption, rather than mFKN.

### CX3CR1-microglia mediated systolic blood pressure improvement in the NTS

**Fig. 3:**
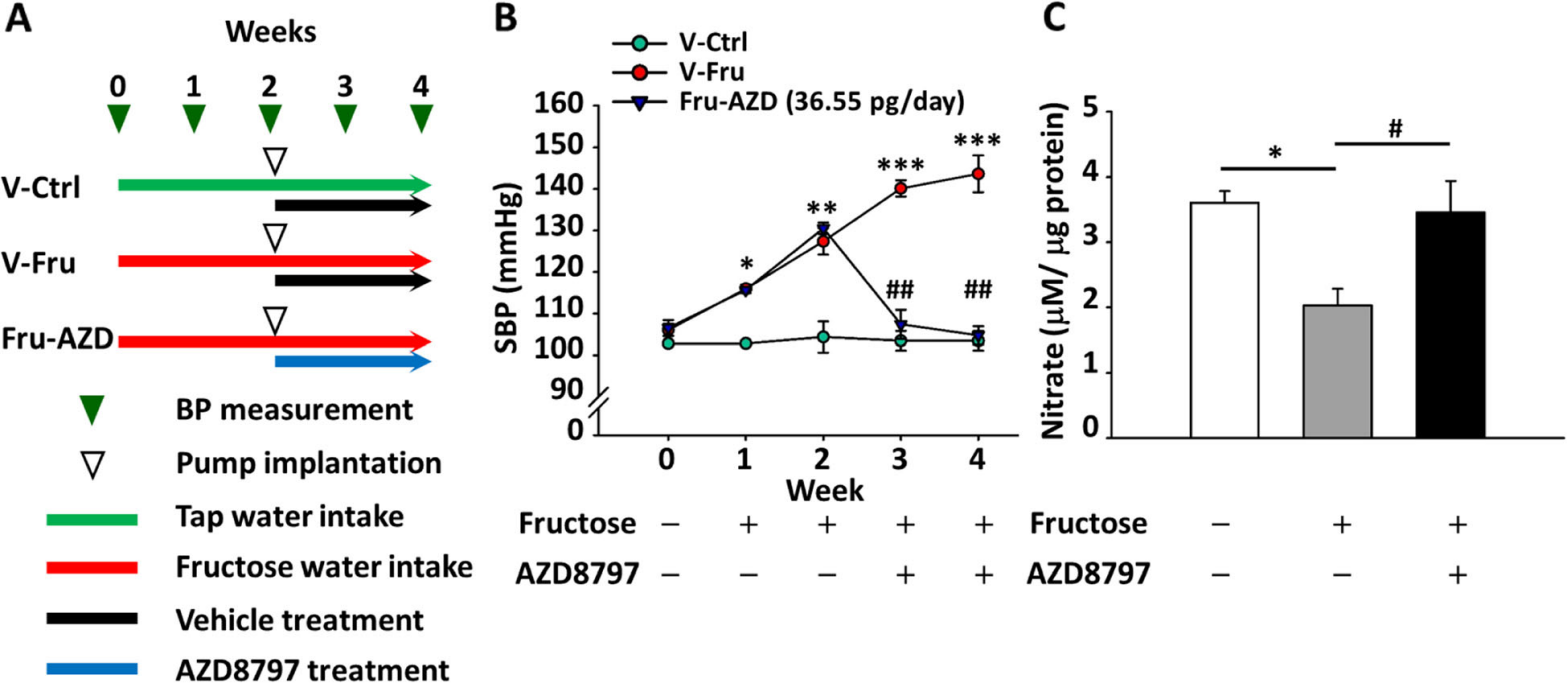
Inhibition of CX3CR1 improves systolic blood pressure and NO production. **a** Time course of systolic blood pressure after intracerebroventricular administration of the CX3CR1 inhibitor AZD8797 for 2 weeks. **b** The bar graph presents NO concentration as micromoles nitrate per microgram of the NTS protein. One-way ANOVA with Scheffe’s post-hoc was performed for statistical analysis. The values are represented as mean ± SEM. *P < 0.05, **P < 0.01, ***P < 0.001 compared to the control group. #*P* < 0.05, ##*P* < 0.01 compared to fructose-fed rats (*n* = 6~8 per group)

To investigate whether CX3CR1-microglia was involved in systolic blood pressure regulation in fructose-induced hypertensive rats, CX3CR1 was inhibited by AZD8797 treatment. AZD8797 was injected into central intracerebroventricularly via osmotic minipump 2 weeks after hypertensive animals were established (Fig. 3a). Furthermore, the hypertensive animals treated with AZD8797 showed systolic blood pressure decreased to normotensive level 2 weeks after treatment began (Fig. 3b).

Our previous studies suggested that fructose-fed rats had reduced NO production in the NTS [21, 22]. Therefore, we measured the NO level and found that NO production was improved in the NTS of AZD8797-treated animals. This suggests that CX3CR1-microglia in the NTS participates in NO and systemic blood pressure regulation (Fig. 3c). The increased NTS microglia activation may be involved in cardiovascular regulation that elicits a pressure effect and attenuates NO production through CX3CR1-microglia activation.

### AZD8797, CX3CR1 inhibitor, restored nNOS pathway in the NTS

**Fig. 4:**
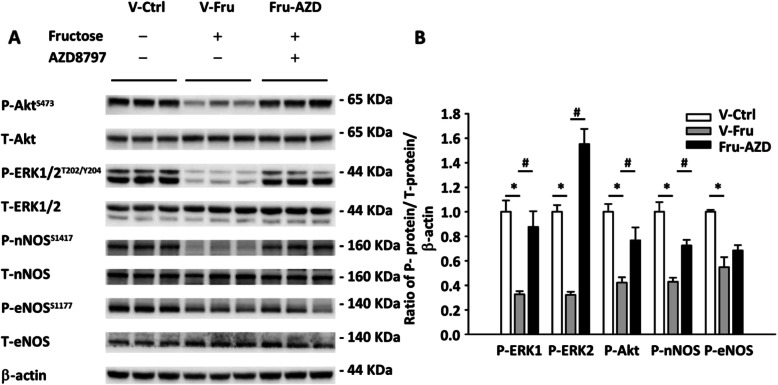
CX3CR1 inhibits the Akt-nNOS pathway in the NTS of fructose-fed hypertensive rats. **a** Semi-quantitative immunoblot analysis determined the phosphorylation level of ERK1/2, Akt, nNOS, and eNOS protein in the NTS of fructose-fed rats. **b** The bar graph shows phosphorylated ERK1/2^T202/Y^204, Akt^S473^, nNOS^S1416^_,_ and eNOS^S1177^ in the NTS of hypertensive rats. One-way ANOVA with Scheffe’s post-hoc was performed for statistical analysis. The values are represented as mean ± SEM. *P < 0.05 compared to the control group. ^#^*P* < 0.05 compared to fructose-fed rats(*n* = 6~8 per group)

It was previously reported that the cardiovascular regulation effect is mediated by Akt signaling in normal rats [13]. However, our previous study showed that fructose intake causes defect of the PI3K–Akt–nNOS pathway [6, 21]. To test the pathophysiological role of CX3CR1-microglia in the fructose-fed rat, we analyzed the Akt–nNOS pathway in the NTS using immunoblotting. The level of phosphorylated ERK1/2, Akt, and nNOS proteins may be recovered after AZD8797 treatment, and phosphorylated eNOS protein did not show any significant difference (Fig. 4). This result suggests that CX3CR1-microglia may be able to inhibit Akt–ERK1/2–nNOS in the NTS of fructose-induced hypertensive rats.

### AZD8797 treatment decreases the pro-inflammatory cytokines in the NTS

To investigate the effect of AZD8797 on chronic pro-inflammatory environment in the NTS, we analyzed the levels of IL-1β, IL-6, TNF-α, and fractalkine expression in the NTS by ELISA. The pro-inflammatory cytokines IL-1β, IL-6, and TNF-α were decreased in the AZD8797 treatment group. The level of fractalkine was not changed significantly after treatment compared to the control (Table 2). These results indicate that AZD8797 improved the inflammatory environment through CX3CR1-microglia inhibition without fractalkine involvement.

**Table 2:** General characteristics of the fructose-induced WKY rats and AZD8797-treated group

Parameter/group	V-control	V-fructose	Fru-AZD8797
NTS fractalkine, ng/mg	5.33 ± 0.43	8.07 ± 0.29*	9.52 ± 0.66^#^
NTS IL-1β, pg/mg	4.83 ± 0.16	6.93 ± 0.20*	4.73 ± 0.13^#^
NTS IL-6, pg/mg	63.58 ± 4.32	76.87 ± 1.34*	49.70 ± 2.14^#^
NTSTNF-α, pg/mg	8.16 ± 0.78	21.63 ± 2.06*	14.16 ± 0.65^#^

The values of quantitative ELISA of NTS areas for FKN, IL-1β,IL-6, and TNF-α are presented as mean ± SEM. One-way ANOVA with Scheffe’s post-hoc was performed for statistical analysis.

*P < 0.05 vs control group (*n* = 6), ^#^*P* < 0.05 vs fructose group (*n* = 6~8 per group)

**Fig. 5:**
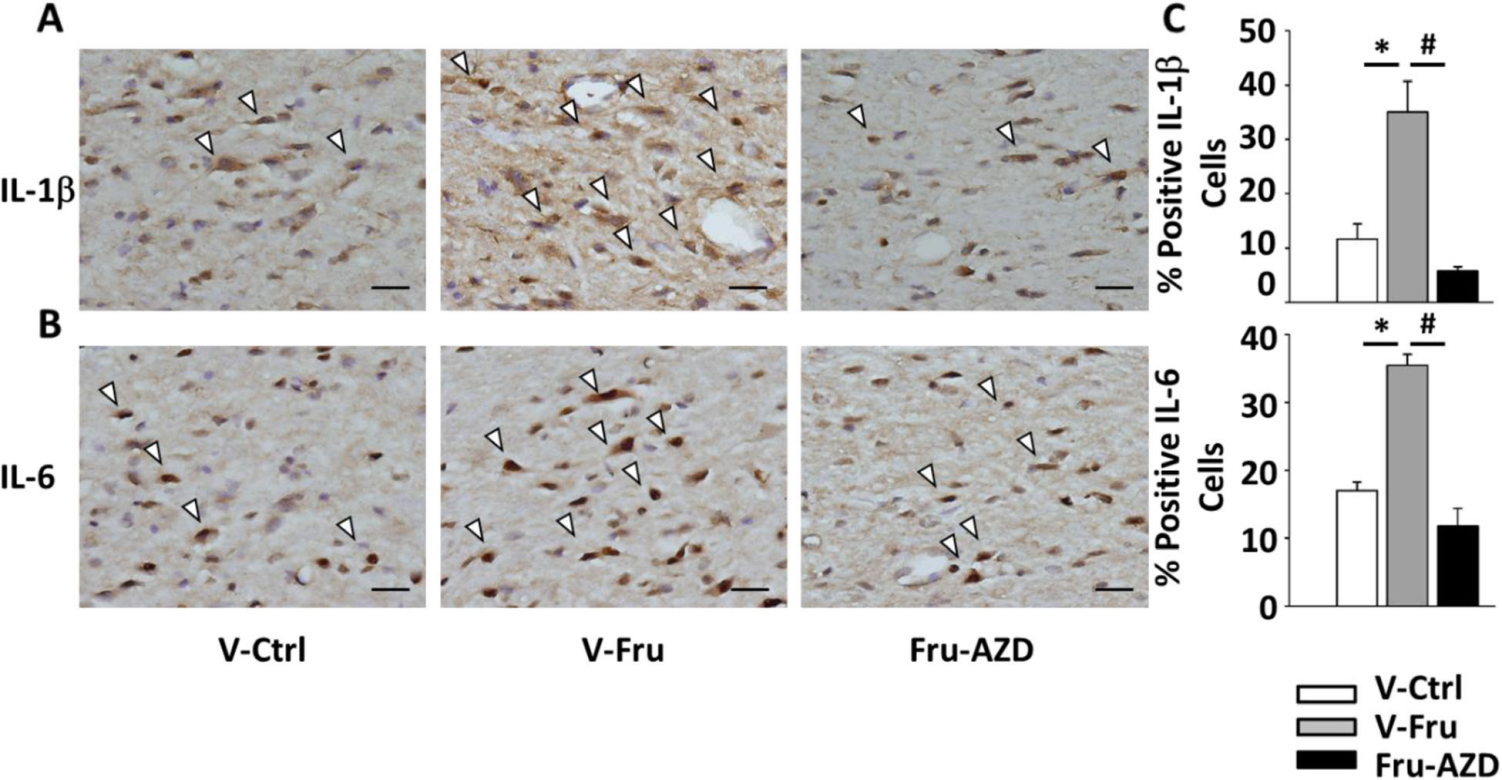
In situ quantitative immunohistochemical analysis of pro-inflammatory cytokines in the NTS following feeding with 10% fructose and AZD8797 treatment. **a** Qualitative analysis of IL-1β and IL-6 were observed by immunohistochemical staining after administration of the CX3CR1 inhibitor AZD8797. The arrowhead indicates positive cells as a representative. The scale bar presents 50 μm. **b** Graphs depicting the quantitative analysis of the in situ cytokine-positive cells. The percentage was determined by counting pro-inflammatory positive cells in each hemisphere of the NTS at 200 × magnification. One-way ANOVA with Scheffe’s post-hoc was performed for statistical analysis. The values are represented as mean ± SEM. *P < 0.05 compared to control rats and ^#^*P* < 0.05 compared to fructose-fed rats (*n* = 6~8 per group)

Distribution of pro-inflammatory cytokines was assessed by immunohistochemistry (Fig. 5a). As expected, AZD8797 treatment decreases significantly released pro-inflammatory cytokines in the NTS (Fig. 5b), suggesting that the role of CX3CR1-microglia is not only in cardiovascular regulation but also in improving the inflammatory environment of the brainstem.

## Discussion

**Fig. 6:**
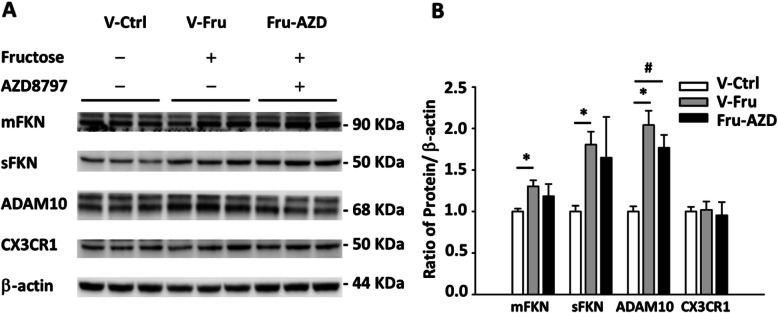
CX3CR1 inhibitor does not affect membrane-anchored FKN (mFKN), soluble FKN (sFKN), and ADAM10 levels in the NTS. **a** Immunoblotting was performed for mFKN, sFKN, and ADAM10 protein expressions in the NTS. **b** The bar graph displays the protein content of mFKN, sFKN, and ADAM10 in the NTS of hypertensive rats. The values represent mean ± SEM. One-way ANOVA with Scheffe’s post-hoc was performed for statistical analysis. *P < 0.05 compared to the control group (n = 6~8 per group)

Although the contribution of the pro-inflammatory factor to hypertension has been increasingly understood, little is known about the functional relation between the pro-inflammatory factor and chronic brain inflammation. In this study, we report that CX3CR1-microglia in the NTS plays a role in fructose-induced hypertension. Inhibited CX3CR1-microglia signaling attenuates fructose-induced hypertension and chronic brain inflammation. The above conclusion is supported by the following observations. ICV administration of AZD8797, a CX3CR1 inhibitor, attenuates fructose-induced hypertension and expression of pro-inflammatory cytokines IL-1β, IL-6, and TNF-α. NO, the gas involved in sympathetic activity and blood pressure regulation in the NTS, was elevated by CX3CR1-microglia inhibition. Furthermore, the pathway important for NO generation, Akt–nNOS, was restored after CX3CR1-microglia inhibition in the NTS of hypertensive rats. The major finding of this study is that CX3CR1-microglia-mediated downregulation of blood pressure and NO production involve the downregulation of the Akt–nNOS pathway and amelioration of pro-inflammatory cytokines in the NTS of hypertensive rats (Fig. 6).

Pro-inflammatory cytokines produced in the brain play a pathophysiological role in the microenvironment to attract leukocyte or monocyte clusters from the blood vessel to the NTS [26]. Their presence in the NTS suggests that pro-inflammatory cytokines might contribute to cardiovascular diseases, such as hypertension [27]. For example, increase in transcription and protein expression of pro-inflammatory cytokines have been reported in the hippocampus after 2 weeks of 60% fructose diet intake [4]. Takagishi et al. reported that IL-6 is an endogenous cytokine in the NTS. Their study proposes that IL-6 microinjection has been reported to have attenuated L-glutamate-induced bradycardia in the NTS [28]. This implies that IL-6 overexpression in the NTS may interrupt cardiovascular regulation through baroreceptor afferents’ signal transmission. Shi et al. demonstrated that IL-1β stimulates blood pressure increase through ICV injection [29]. Our present observations suggest that fructose feeding promotes early chronic pro-inflammatory cytokines IL-1β, IL-6, and TNF-α in the NTS.

The FKN and CX3CR1 is a ligand–receptor pair for neuron–microglia communication. Ruchaya et al. reported that FKN and CX3CR1 are abundantly expressed in NTS, and FKN microinjection in the NTS is concerned with cardiovascular regulation [13]. Their findings support the rationale for investigating CX3CR1’s role in the NTS of the hypertensive animal model that has brain inflammation. For this reason, we selected AZD8797 to inhibit CX3CR1 for investigating its pharmacological role towards inflammation during hypertension.

Our previous study proposed that NOS function in the NTS may regulate cardiovascular function via PI3K–Akt–nNOS and ERK1/2-eNOS pathways [23, 25, 30]. Ruchaya et al. found that FKN regulates cardiovascular function in the NTS through FKN and LY294002 (PI3K–Akt inhibitor) microinjection [13]. AZD8797 is a selective CX3CR1 inhibitor, which has been applied in the therapy of the multiple sclerosis model [11]. We used the ICV method for the delivery of AZD8797. Interestingly, AZD8797 decreases inflammation and increases NO production, which suggests that CX3CR1 in metabolic syndrome is different from its normal state.

FKN is cleaved by metal proteases, such as ADAM10, ADAM17, or MMP-2, which release soluble chemokine domain out of the cells. According to our data, fructose intake induces metal proteases, and the soluble FKN stimulates CX3CR1-expressed cells (Fig. 6). Although this result conflicts with the work previously reported [13], the improvement in inflammation by AZD8797 agrees with the results of Wollberg et al. in that spinal cord inflammation is improved in multiple sclerosis [11].

Previous studies have demonstrated that central inflammation characterizes the hypertensive state and participates in blood pressure elevation [29]. The importance of central inflammation in hypertension progression, mortality modulation, and neuronal degeneration disease is now well established by animal models; a theory that Alzheimer’s disease is type 3 diabetes has recently been proposed [31, 32]. It has been hypothesized that Alzheimer’s disease occurs as a result of decreased NO production in the brain to an elevated, operating pressure [33]. In this study, we investigated the mechanism for CX3CR1-microglia in regards to cardiovascular modulation in the NTS and found that the ERK and Akt cascade, which were initially discovered as essential regulator for cell division and differentiation, in fact, participates in CX3CR1-microglia-mediated central cardiovascular regulation. In addition, we also demonstrate that neuronal nitric oxide synthase (nNOS) was mediated by CX3CR1-microglia, rather than endothelial nitric oxide synthase (eNOS), which was originally identified in the vascular endothelium and participates in cardiovascular regulation in the NTS. Further investigation of the molecular mechanisms involving blood pressure regulation may unravel the pathogenesis of CX3CR1-microglia and brain inflammation in hypertension.

G protein-coupled receptors (GPCRs) play an important role in drug therapy and represent one of the largest families of drug targets for various diseases such as depression and cardiovascular diseases. Previous studies suggested that CX3CR1, a ubiquitously distributed GPCR in the neuron system, participated in blood pressure regulation in the NTS. Insulin resistance–metabolic syndrome patients have been reported to benefit from strategies concerning stimulation of VSMC survival and reduction in FKN/CX3CR1 signaling to promote plaque stability through an IRS2-dependent signaling [34]. We previously proposed that IRS1 is more critical than IRS2 in insulin resistance–metabolic syndrome of the NTS [6, 35, 36]. This study suggests that the inhibition of CX3CR1-microglia improves the defective Akt and ERK1/2 signaling, both of which are critical pathways for NO production in the NTS. Currently, it potentially appears that improvement in insulin resistance is mediated byCX3CR1-microglia in hypertension progression.

**Fig. 7:**
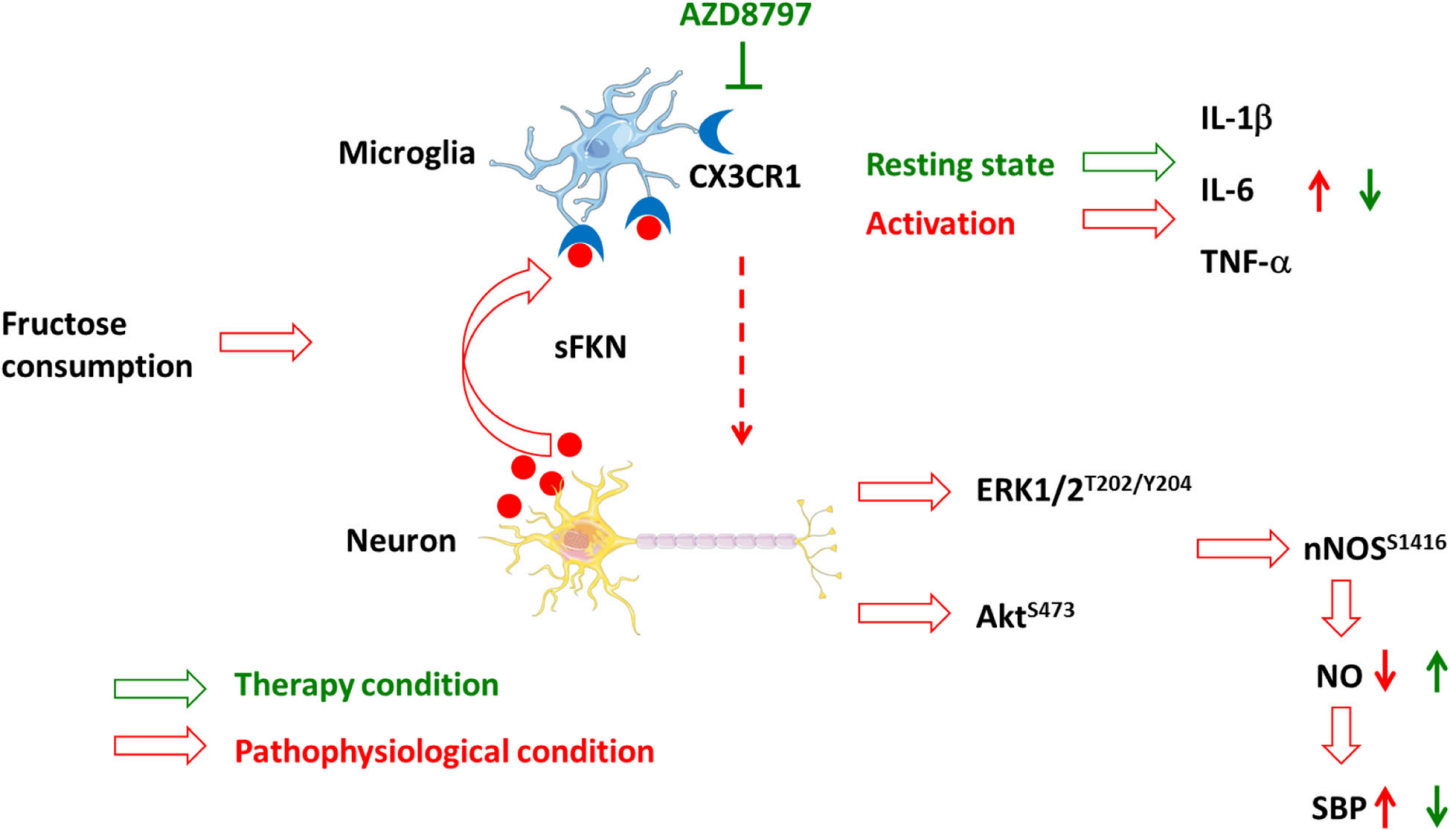
Proposed mechanism for CX3CR1-microglia and fractalkine during hypertension triggered by fructose in the NTS. Treatment with a CX3CR1 inhibitor (AZD8797) illustrated that CX3CR1-microglia acts as regulator of blood pressure through the ERK1/2-Akt-nNOS pathway. Inhibition of CX3CR1 decreases blood pressure and enhances the activity of Akt-ERK1/2-nNOS pathway in the NTS during fructose-induced hypertension

In summary, we showed that AZD8797, a CX3CR1 inhibitor, attenuated fructose-induced hypertension and expression of pro-inflammatory cytokines, IL-1β, IL-6, and TNF-α. AZD8797 also elevated NO generation, the gas molecule involved in sympathetic activity for regulating blood pressure in the NTS. The CX3CR1-microglia inhibition in turn repaired the Akt–nNOS pathway, an important pathway for NO generation in the fructose-induced hypertensive rat (Fig. 7).

## Conclusion

This study provides the fundamental role of CX3CR1-microglia, which can be used for treating cardiovascular disease because these receptors are involved in various pathological conditions. Our novel findings suggest that CX3CR1-microglia may be a potential candidate for treating hypertension and relieving autonomic nerve function through reestablishment of normal crosstalk between the neuron and microglia.

## Data Availability

All data generated or analyzed during this study are included in this published article.

## Abbreviations

BP: Blood pressure
CSF: Cerebral spinal fluid
CX3CL1: C-X3-C motif chemokine ligand 1
CX3CR1: C-X3-C motif chemokine receptor 1
dHDL: Direct high-density lipoprotein
FKN: Fractalkine, another term of CX3CL1
HP-β-CD: 2-hydroxypropyl-β-cyclodextrin
IL-1β: Interleukin-1β
IL-6: Interleukin-6
TNF-α: Tumor necrosis factor-α
ICV: Intracerebroventricular injection
mFKN: Membrane-anchored fractalkine
NO: Nitric oxide
nNOS: Neuronal nitric oxide synthase
NTS: Nucleus tractus solitarii
SBP: Systolic blood pressure
sFKN: Soluble fractalkine
WKY: Wistar-Kyoto rat
